# Exploring the perspectives and experiences of food-insecure adults who are also tobacco dependent: a qualitative study in North East England

**DOI:** 10.1136/bmjph-2025-002683

**Published:** 2025-10-17

**Authors:** Kerry Brennan-Tovey, Nida Ziauddeen, Laura Hamilton, Carol Gorman, Eileen Kaner, Darren McMahon, Susan Jean Mountain, Sheena E Ramsay, Claire Thompson, Maria Raisa Jessica Aquino

**Affiliations:** 1 Newcastle University Population Health Sciences Institute, Newcastle upon Tyne, UK; 2 University of Southampton School of Primary Care Population Sciences and Medical Education, Southampton, UK; 3 University of Hertfordshire Centre for Research in Public Health and Community Care, Hatfield, UK; 4 Public Member, Newcastle, UK

**Keywords:** Public Health, Sociodemographic Factors, Qualitative Research

## Abstract

**Abstract:**

**Introduction:**

Smoking and tobacco dependence remain the single biggest preventable cause of death and illness in England. A disproportionally high number of adults using tobacco products also experience poverty and food insecurity. Those in the lowest socioeconomic groups are both more likely to use tobacco and more likely to experience food insecurity. Due to the ‘cost-of-living-crisis’, there is a need for more qualitative research in tobacco dependence and food insecurity. Yet, there is a lack of qualitative UK-based research examining how these two issues are linked for the people experiencing them. This study explores the perspectives and experiences of tobacco dependency among adults who also experience food insecurity.

**Methods:**

Semi-structured interviews were conducted with adults who were experiencing food insecurity and identified as tobacco dependent in 2023. 19 participants (female n=12) were recruited from two North East England food aid organisations. Interviews explored previous and current patterns of tobacco use, experiences of food insecurity and of budgeting personal finances. Data were analysed using thematic analysis.

**Results:**

We identified three themes in the following topic areas: how participants planned and structured their tobacco dependence, the financial constraints of tobacco dependence among food insecurity and how they ensure the ability to afford purchasing tobacco products, and the perceived advantages and disadvantages of tobacco dependence.

**Conclusion:**

To conclude among people who were food insecure, tobacco use was strongly influenced by their circumstances and the social and material stressors they face. These aspects are potentially important considerations when developing smoking cessation support for people who are food insecure.

**WHAT IS ALREADY KNOWN ON THIS TOPIC**

- Smoking prevalence is decreasing among nearly all populations within the UK; however, those living in lower socioeconomic groups are less likely to succeed in a quit attempt. Previous studies have shown a strong link between those living with high levels of food insecurity and high levels of tobacco dependency, but a lack of studies has explored the experiences of this population, and in a UK context.

**WHAT THIS STUDY ADDS**

- The first study to provide insight into the complexity of tobacco dependence among food-insecure adults in the UK. Providing understanding of tobacco use as a form of stress relief and as an appetite suppressant when unable to purchase enough food, how individuals budget financially for tobacco and food including their perception on the importance of both. Tobacco dependence and quitting was framed as an individual concern and not that of the household, whereas items such as food, bills and housing were.

**HOW THIS STUDY MIGHT AFFECT RESEARCH, PRACTICE OR POLICY**

- Our findings provide awareness into the experiences of those who feel unable to engage with local tobacco cessation service and how food insecurity denotes an additional layer of deprivation and problematises the already difficult prospect of quitting. Providing policymakers with the evidence to allow them to consider factors such as the use of tobacco as an appetite suppressant, a form of stress relief and the financial cost as a motivator to quit, when developing smoking cessation services for this population.

## Introduction

 Smoking and tobacco dependence remain the single biggest preventable cause of death and illness in England. In 2018, 14.7% of adults in the UK smoked cigarettes,^1^ with disproportionately higher rates of smoking for population groups with poor educational attainment, mental ill health, manual occupations, living in social housing and experiencing poverty or homelessness.^2^ Socioeconomic differences associated with smoking behaviours have become one of the major contributors to health inequalities, including quality of life, morbidity and mortality.^3^ Another aspect of poverty and disadvantage associated with smoking is food insecurity.^4^,^6^ Food insecurity is defined as ‘*The inability to acquire or consume an adequate quality or sufficient quantity of food in socially acceptable ways, or the uncertainty that one will be able to do so*’.^7^,^10^ The ‘cost-of-living crisis’ in countries such as the UK has further exacerbated household food insecurity.^11^,^14^

Smoking and socioeconomic status are strongly linked.^15^ In the UK, smoking prevalence has been declining since the 1970s among all socioeconomic groups; however, there has been no significant narrowing of the gap between routine and manual socioeconomic groups,^16^ making tobacco use a major health disparity issue.^17^ In the UK, quit rates are higher among those in higher socioeconomic groups,^18^ with previous research finding that 80% of smokers experienced some form of disadvantage.^19^ While smokers in routine and manual occupational groups try to quit as often as their peers in professional and managerial groups, they are less likely to succeed.^20^ This has been linked to social factors (such as stress, boredom and a lack of social support)^21^ and the level of dependence on nicotine (ie, they start smoking earlier in the day, smoke more cigarettes per day^22^ and consume more nicotine per cigarette than the most affluent smokers).^23^

Evidence^24^ has shown that smoking cessation services tend to result in higher quit rates among higher socioeconomic status smokers and are therefore unlikely to reduce inequaflities in smoking; however, other studies^24 25^ have shown that cessation services can be effective for smokers in lower socioeconomic groups by concentrating the support in less advantaged communities. Evidence^25^,^27^ has shown that some smoking cessation services have been successful in attracting smokers from deprived areas; however, they are significantly less likely to have a successful quit. One study^28^ found that cessation rates were lower in more deprived areas (52.6%) compared with all other areas (57.9%), but the cessation rate was higher as a proportion of all smokers treated (8.8% and 7.8%, respectively). Smokers from lower socioeconomic groups face a number of challenging barriers to quit, including high levels of dependence,^15^ high levels of stress^29^ and pro-smoking community and society norms,^30^ with very few smoking cessation programmes considering the difficulties of those in lower socioeconomic groups.^31^ Lower socioeconomic groups often face poverty, economic insecurity, housing insecurity or homelessness, poor education, caregiving responsibilities, poor mental health and low self-efficacy.^30^

Food insecurity can drive individuals to use substances like tobacco as a coping mechanism for both emotional distress and the physical experience of hunger. Nicotine’s appetite suppressing properties may make smoking a strategy for those with limited food access.^6 32^ Consequently, restricted food intake can reduce individuals’ ability to resist smoking urges, increasing dependence.^33^ Some studies^6 34 35^ have shown that mothers forego their own nutritional needs to ensure their children are fed, sometimes turning to smoking to deal with the stress and discomfort of personal food deprivation. Concerns about post-cessation weight gain, which can be particularly relevant in populations already vulnerable to obesity due to food insecurity, may also discourage attempts to quit.^36^

In Germany, it was found that across all (state levels) food insecurity severity levels, smokers appeared to be more often food insecure than non-smokers.^37^ Some studies report that smoking limits available financial resources that could be spent on food,^38^ while other studies state that being food insecure increases the likelihood of smoking, as it might be used as a coping strategy.^28^ A further study in Canada found that 49% (n=240) of food bank users (n=490) within the study smoked, with the mean number of cigarettes smoked per day being 20.2,^39^ and an additional study in the USA also showed that 50% (n=64) of food assistance recipients were current smokers.^40^

This study aimed to explore the perspectives and experiences of adults (18+) who are tobacco dependent and experiencing food insecurity, regarding their health, tobacco dependence and available smoking cessation services, in order to gain insight into the specific difficulties this population faces when attempting to quit.

## Methods

### Research design

A qualitative study using semi-structured interviews with adults experiencing food insecurity who self-report as smoking tobacco products (ie, cigarettes, rolling tobacco or chewing tobacco). All recruitment and data collection were carried out by the first author.

### Patient and public involvement

Four public members with lived experience were involved in the funding application. Three remained involved in the study and assisted in developing the study documents (ie, information sheet, consent form, topic guide and recruitment posters), providing input into the language used to reduce any stigmatising language. Once preliminary themes were developed, a workshop was conducted with the first author and three public members, who provided input in the refining and naming of themes.

### Participation recruitment

A purposeful sampling method was used to recruit participants to interviews from two food aid organisations in the North East England using the following eligibility criteria: (1) aged 18 years or over, (2) living in the North East England, (3) self-identify as experiencing food insecurity and (4) self-identify as a smoker of tobacco products (currently or within the last 12 months). Recruitment took place between May and November 2023. Recruitment posters were publicly displayed in both premises, shared directly on their social media pages, and a researcher visited both locations in-person to recruit participants. Potential participants either phoned or emailed the first author to express interest or could speak with the first author when they were attending the food aid organisations. Participants were provided with a study information sheet, and a short discussion with the first author and informed consent was sought before taking part.

### Data collection

One-to-one semi-structured interviews (n=19) were conducted either face-to-face within the food aid organisation, via telephone or videoconferencing software (ie, MS Teams/Zoom), until data saturation was reached, at which no new information, codes or themes were collected from the data.^41^ A bespoke topic guide (online supplemental file 1) informed by existing literature,^6 42^ discussions with the research team and public members, was pilot tested and used to gather experiences and perspectives of adults who experience food insecurity and smoke tobacco products. Topics included history and patterns of tobacco use; current and past experiences of food insecurity; coping strategies for life stressors; thoughts and experiences of quitting tobacco use; and budgeting personal finances. Interviews were audio recorded and transcribed verbatim. Interviews last between 15 and 40 min. Participants received a £20 voucher as reimbursement for their time. Recorded audio files were transcribed verbatim, and a member of the research team checked transcriptions for accuracy and anonymised participant details.

### Data analysis

Interview data were managed using NVivo software^43^ and analysed thematically^44^ using an inductive analysis method which allowed for amplifying participants’ experiences. Data analysis followed Braun and Clarke’s six-phase approach.^44^

Transcripts were coded by the first and second authors. Initially, two transcripts were independently coded by both authors, which were discussed and used to develop a codebook. The remaining transcripts (n=17) were then independently coded by first (n=10) and second (n=7) authors, with regular meetings taking place to discuss initial findings and update the codebook. Previously coded transcripts were reviewed and recoded, if needed, by first or second author when edits were made to the codebook. Any coding disagreements or discrepancies were resolved between the first and second author. Themes were developed by the first author, with input from the second author and reviewed by the research team and public members.

## Results

### Participant characteristics

19 participants, n=14 recruited from first food aid organisation, n=5 from the second food aid organisation, participated. No participants withdrew from the study.

Table 1 provides the demographic information for participants.

**Table 1:** Participant demographic information

Characteristics	N (%)
Sex	
Female	12 (63.2)
Male	7 (36.8)
Ethnicity	
White British	17 (89.4)
White—other	1 (5.2)
Black British	1 (5.2)
Age group (years)	
21–34	5 (26.3)
35–49	9 (47.4)
49–64	4 (21.0)
65+	1 (5.3)
Form of tobacco use	
Rolling tobacco	12 (63.2)
Cigarettes	4 (21.0)
Vape	0
Combination tobacco/vape	3 (15.8)
Employment status	
Employed	5 (26.3)
Unemployed	8 (42.1)
Self-employed	1 (5.3)
Volunteer	2 (10.5)
Retired	3 (15.8)
Housing status	
Homeowner	1 (5.3)
Rent—social/council housing	9 (47.4)
Rent—private	6 (31.5)
Emergency or temporary housing	3 (15.8)
State benefit recipient	
No	2 (10.5)
Yes	17 (89.5)

### Thematic results

We identified three overarching themes from the thematic analysis of interview data in the following areas—patterns of tobacco dependence among food insecurity, financial constraints of tobacco dependence and food insecurity and perceived health effects and trade-offs of tobacco dependence and food insecurity.

#### Theme 1: patterns of tobacco dependence among food insecurity

This theme focuses on participants planning and structuring their tobacco dependence among the stress of everyday life and living with food insecurity. This theme included two sub-themes: (1) smoking habits and stressors and (2) experiences of attempts to quit.

##### Smoking habits and stressors

Smoking occupied a central role in people’s lives that both helped structure their day and cope with stressors. Those participants who were in employment explained how they thought and planned ‘smoking breaks’ while at work, “*If I’m at work, then I try and get one at least an hour*” (male, aged 30–39 years old). Often the number of cigarettes smoked in a day was limited. One participant describes limiting their cigarettes, “*I wouldn’t say more than 12 or 13. I do limit myself*” (female, aged 50–59 years old).

Two forms of stress were identified that impacted upon the uptake and continuation of smoking: extremely stressful life events and common everyday stressors. Traumatic life events included things like the sudden and critical illness of a child or loved one or navigating the court system, “*I had packed in smoking when my dad died, and then I just couldn’t cope any longer*” (female, aged 50–59 years old). Common everyday stressors included financial constraints, ensuring enough food is available, housing instability and inability to secure employment impacting their smoking. In these stressful situations, smoking was reported as a form of stress relief, citing that they felt it reduced stress levels and provided a calming effect, “*It helps a lot just because if I get that stressed, I have to go and have a tab and then I can just feel my, I don’t know what it is I just feel myself calming down. That’s the only way I can really explain it”* (female, aged 30–39 years old). These everyday stressors are also common drivers of food insecurity. Experiences of tobacco dependency in the context of food security are complex, with tobacco sometimes taking the place of food. For example, smoking was reported by many as a morning priority and was also accompanied by other morning activities such as with a hot drink (often coffee or tea), and usually before food, or in some cases instead of breakfast. “*I have my next smoke maybe before I want to have my breakfast and after. Most times if I don’t have access to food, I’ll see the smoke as my breakfast”* (female, aged 20–28 years old).

Tobacco use was seen as an individual concern, a task that they completed on their own, “*I wouldn’t, I don’t with food but once I’ve had food and everybody’s finished, I would go to the back like into the kitchen, have the back door open and have a tab then”* (female, aged 30–39 years old)*,* with one participant reporting that smoking was, “*It’s that one vice”* (female, aged 50–59 years old), while another reports, “*I used to do it in private”* (female, aged 40–49 years old). Whereas food and meals were considered more of a household and family activity, “*We eat breakfast whether it’s together or whether they just eating by themselves”* (female, aged 30–39), and “*teatime is the time that we are talking about our days”* (female, aged 30–39 years old). Others reported relying on family for cooked meals, “*I rely on my parents for cooking and that to be quite honest”* (male, aged 30–39 years old), and going food shopping together as a family, “*Tesco is just up the road about, well it’s 10-minute walk for me now. Asda I have to go to [NORTH EAST TOWN], so I would have to travel on a bus or a taxi …. my husband has a bus pass but he goes down on his mobility scooter, so I just meet him at [NORTH EAST TOWN] but my daughter always comes with us because she’s got a bus pass as well”* (female, aged 60–69 years old), or “*Then it’s normally a Thursday once a fortnight, my son comes with us to Asda and helps us do a bit of a shop”* (female, aged 50–59 years old).

##### Experiences of attempts to quit

Previous quit attempts were frequently reported by participants. One participant had quit for 6 years due to pregnancy. Others had attempted to quit due to a dislike of smoking, their knowledge of the health implications and some due to financial constraints of tobacco purchasing, “*I suppose it’s maybe been when I’ve been quite broke and thought that’s going to be the cheaper option”* (female, aged 40–49 years old). Many participants described the different resources and services they had used during previous quit attempts. These included forms of nicotine replacement therapy (NRT), such as nicotine patches, inhalators, nicotine chewing gum, nicotine pastels and medications (ie, Zyban, a brand of bupropion). One participant expressed how one form of NRT had worked well for them in a previous quit, “*And I’d been and getting some patches and I put a patch on in the morning and by the afternoon I’d took the patch off and I never had another cigarette for seven years*” (female, aged 60–69 years old). When reflecting on if they had a desire to stop smoking in the present, many had no desire to quit smoking, stating they felt that their stress would not be managed, highlighting the belief that tobacco products reduced stress, or allowed them to ‘cope’, “*I’m not at the point where I know I would be rather worse off if I would stop smoking tomorrow because the stress of not being able to smoke again and the stress of daily basic life would be rather greater”* (female, aged 30–39 years old).

### Theme 2: financial constraints of tobacco dependence and food insecurity

This theme focuses on the financial constrictions of tobacco dependence, how participants implemented strategies to ensure the ability to afford and purchase tobacco products while ensuring they had enough food for themselves and their families. This theme is made up of three sub-themes: (1) budget management; (2) strategies used to extend access to tobacco products and (3) strategies used to extend food.

#### Budget management

Many participants reported budgeting their finances on a monthly basis and explained how food featured in this. Specifically, they explained that there is a hierarchy of spending commitments, prioritising larger bills and utilities at the beginning of the month (including rent, gas, electricity and water) while nominal bills (including phone bill) would not be paid if there were financial difficulties, “*But there has been times where certain thing hasn’t got paid because I haven’t had the money in so I’ve ended up with a little bit of problems with the bank”* (male, aged 40–49 years old). It was reported that once bills had been paid the remainder of their finances would be split to buy food and other required items:

Always at the top I have like my rent and obviously my gas and electric, obviously other bills and I know what I’ve got left so I know what to, how much I can use for food once all they’re paid out because obviously, I know how I work. Now I’ve got myself in a routine. And whatever’s left, basically half it. Sometimes I’m left with like 800 and half of that or just under half of that would be food and that’s like fridge, cupboards, freezers, everything. (Female, aged 30–39 years old)

Some reported that food was a priority over tobacco, “*I always, if I have something left that’s when I buy cigarettes. They are not priorities. The groceries are priority”* (female, aged 30–39 years old), or “*eating comes before the smoking”* (female, aged 20–29 years old), while others perceived tobacco products the priority over food, “*I think if I had to put the level of priorities, it would have to be, the rent is obviously a priority first. And then the council tax. And then it would be the rollies, like the tobacco”* (male, aged 30–39 years old). As a result, participants deployed a range of strategies to cope and prioritise spending, which they explained as specific to either tobacco or food, even though these strategies contained similar approaches.

#### Strategies used to extend access to tobacco products

Several strategies were used to ensure that tobacco products lasted as long as possible. Strategies included limiting the number of tobacco products smoked per day or smoking half of the tobacco product and saving the other half for later:

Usually what I’ll do is obviously I’ll make the roll up and I never ever smoke the full roll up in one go. I always put it out and then obviously when I fancy another one I’ll just light up what I’ve put out. I’m not actually smoking a full one in one go. (Female, aged 20–29 years old)

It was commonly reported that borrowing either money or tobacco products from friends or family was used as a final resort should the participant have no access to tobacco products or money; however, they felt it was important to always pay this back:

I mean my mam [Sic] and dad, they kind of help me when they can, but they are on the very minimum, because they’re in the sixties now. And they sometimes help me out, but it’s only on the basis that I can then, kind of, pay them back or at least help them out when they need it. (Male, aged 30–39 years old)

The most commonly reported strategy was the purchasing of illegal tobacco, which was reported to be approximately 50% cheaper than purchasing legal tobacco products. This was appealing to participants as it made it more affordable for them to continue smoking their desired amount of tobacco. “*The reason is it is half the price in their house than what it is in the shop. Literally half the price, otherwise I’d be smoking a hell of a lot less, to be honest”* (female, aged 50–59 years old). Illegal tobacco was often sourced through an acquaintance or a friend and was frequently a tobacco brand that could not be bought legally within the UK, “*You can’t buy Turner in UK supermarkets, they don’t sell it?”* (male, aged 40–48 years old). However, there was a small minority of participants who were opposed to illegal tobacco, citing that it was more harmful due to it not being regulated:

I think it’s worse than the stuff you buy from the shop because it’s like the illegal one you don’t know what’s in it, mainly. But at least if it’s, you buy it from the shop you know it’s been approved and stuff like that so you’re guaranteed it’s not going to be as bad as the illegal stuff. (Female, aged 20–29 years old)

#### Strategies used to extend food

Similarly, a number of strategies were used to ensure that food lasted longer and included portioning food, eating small portions, batch cooking and freezing meals, meal planning, eating leftovers, skipping meals or eating one meal per day. Budget supermarkets were often used due to the availability of cheaper food options:

Thursday once a fortnight, my son comes with us to Asda and helps us do a bit of a shop, but it’s only basic, it’s not a massive shop, it’s a basic shop really, like bread because I do like toast, I like my toast, milk and my drinks, my juices and my water. (Female, aged 50–59 years old)

Participants also reported ‘shopping around’ to source food for the cheapest price, often purchasing supermarkets’ own brand food items, “*I try and buy the cheapest as possible”* (female, aged 40–49 years old).

Shopping at larger supermarkets was also popular due to the discounts and savings on food items that store loyalty or reward cards provided. While it was often desirable to shop at budget supermarkets, most participants only had access to public transport and they were usually difficult to travel to on foot, resulting in an increased travel cost on public transport, “*Well, my husband has a bus pass but he goes down on his mobility scooter so I just meet him at [North East Town] but my daughter always comes with us because she’s got a bus pass as well”* (female, aged 60–69 years old).

All participants had experience of using food aid to ensure that they had sufficient levels of food and reported that they had had positive experiences with food aid in the past. However, the use of food aid organisations was often seen as a last resort and a ‘top-up-shop’ to the food that they had already purchased:

Yeah, when I’m running out of the food I’m just going to the foodbank and then I’m kind of like mix and match whatever I have left from the last grocery shopping, if you know what I mean. That’s like, foodbank is basically topping up or just having that possibility to cook something cooked for that day when I’m picking the food parcel up. (Female, aged 30-39 years old)

### Theme 3: perceived health effects and trade-offs of tobacco dependence and food insecurity

This theme focuses on the perceived advantages and disadvantages of smoking tobacco.

Participants reported having a strong dislike of smoking, stating they disliked the smell, the financial cost, the nicotine staining teeth and/or fingers, and the negative impact smoking has on their health, “*Everything. The smell. The damage it’s causing to your insides and stuff. You can get some very sore throats. I don’t like that. I get chest pains off it. I just don’t like any of it”* (female, aged 20–29 years old). It was well understood among participants that smoking negatively impacted their health, especially respiratory illnesses such as asthma, chronic obstructive pulmonary disease and emphysema, or resulting in a cough, increased shortness of breath, which impacted activities of daily living, dental concerns, bad breath and poor taste, “*It affects my chest horrendously, really bad, that’s why I hate smoking”* (female, aged 50–59 years old). A large number of participants were able to describe where their nearest smoking cessation services were and how to access them (ie, general practitioner, specialist stop smoking nurse and local pharmacist). While those participants who also acknowledged they smoked cannabis alongside tobacco products reported concerns around receiving help to stop smoking both substances, “*Because I’d obviously have to stop green as well which is the hardest part of that”* (male, aged 20–29 years old). Participants showed extensive knowledge of NRT available to them, often citing previous use, and often a preference, “*I’ve tried patches, I’ve tried the tablets, I’ve tried the small inhaler and I’ve also tried vaping, but that’s over the last ten years, it’s been sporadic, I’ve never stuck”* (female, aged 40–49 years old).

However, participants also reported several perceived advantages to smoking relevant to managing food insecurity, including suppressing appetite resulting in easing skipping meals, avoiding eating and losing weight, helping reduce high levels of stress, increasing calmness and reducing anxieties. It was commonly reported that smoking tobacco products also bought a level of enjoyment, “*I mean I smoke because I enjoy it. It’s not because I choose to have food or whatever. I enjoy smoking. It sounds funny but I do enjoy smoking”* (female, aged 60–69 years old). Often believed to be a task that is done solely for the participant, “*Most of the time I like to smoke. I suppose for me it’s because it’s the only thing I’m in control of, which is a bit stupid to say”* (female, aged 40–49 years old). Alongside this, a small number of participants focused on the idea that smoking helps them lose weight, through reducing their appetite, or substituting meals for smoking tobacco, “*Because smoking takes away my appetite. I mean, I’ve lost weight since I started smoking. It takes my appetite away so instead of eating sometimes, if I’m at work and hungry instead of pulling over and having a sandwich, bag of crisps which takes a bit time, I’ll just have a quick two min tab”* (male, aged 30–39 years old).

## Discussion

This study provides insight into tobacco dependence among food-insecure adults in North East England. Participants reported relying on tobacco use as a form of stress relief due to prolonged highly stressful life events (ie, financial instability and inability to purchase enough food). While participants in this study generally understood the health implications of tobacco use and had a dislike of tobacco use, they felt that tobacco use had an advantage to them, in that it reduced their stress and anxieties. Participants described similar pressures affecting their ability to access food and tobacco and similar approaches to managing their spending on them. However, they framed tobacco use and quitting as individual concerns, separate from household provisioning issues like food, bills and housing. The experience of tobacco dependence in the context of food insecurity is a complex one.

### Contributions to the literature

This article offers understanding into the experiences of those who do not choose to or feel unable to engage with local tobacco cessation services. While previous studies^4^,^629 37^ have largely been quantitative or explored the experiences of tobacco use among food-insecure adults in the USA, this was the first study to explore the experiences of this population within a UK context. Findings which suggest that tobacco can reduce anxiety and stress correspond with previous research.^28^ As do accounts of it being used to cope with food insecurity by reducing appetite. Previous studies^6 29^ reported finding similar responses in a US population which reported a physiological effect of nicotine is appetite suppressant^32^ and nicotine withdrawal has been associated with increased eating.^45^ Participants of this study relied on tobacco as a form of appetite and hunger suppression. Strategies to afford both tobacco and food, including the purchasing of illegal tobacco, reducing the amount smoked, borrowing tobacco and/or food, skipping meals,^46 47^ and the use of food aid organisations, have been described elsewhere,^5 48^ and build upon the idea that smoking limits available resources that could be spent on food.^38^

Tobacco-related expenditures have been shown to exacerbate financial strain by competing with spending on household necessities, such as food.^49^,^52^ Interestingly, the participants of this study framed their relationship with tobacco and attempts to quit in individual terms, and their struggle to afford food as a household or community issue. Doing this, arguably, had the effect of creating discursive distance between food and tobacco, going some way to negate implied concerns that the speakers were spending money on tobacco that could be spent on food. Such framings are not uncommon in the accounts of smokers^53^ and need to be factored in to efforts to support people to quit.

### Implications

Tailored interventions are required to support quitting tobacco among food-insecure groups. Food insecurity denotes an additional layer of marginalisation and deprivation that further problematises the already difficult prospect of quitting. The findings of this study suggest that an important first step could be helping individuals to integrate their tobacco and food budgets and, thereby highlight the full financial cost to them as a powerful motivation for ceasement. Future interventions would need to consider the implications of this population viewing food as a household concern, whereas they view their smoking and quit attempts as an individual while considering the implications of tobacco products on reducing appetite and perceived stress reduction to ensure that those who are food insecure have access to support and additional food resources during quit attempts.

### Strengths and limitations

A strength of the study was that it is the first to qualitatively explore the experiences of adults who are both tobacco dependent and food insecure in a UK context. A limitation of the study was that it did not further explore the barriers faced when making a quit attempt. Additional work is required to determine why this population is not ready to engage in a quit attempt and explore the barriers that are preventing them from engaging with tobacco dependence services. Another limitation was the lack of diversity within the sample. Unfortunately, it was difficult to recruit a diverse population from within the two food aid organisations, whose service users are predominantly white British. Further work is required to ensure ethnic diversity.

## Supplementary material

10.1136/bmjph-2025-002683 online supplemental file 1

## Data Availability

No data are available.
