# Supplementary material for: Exploring the perspectives and experiences of food-insecure adults who are also tobacco dependent: a qualitative study in North East England
Source: BMJ Public Health. 2025 Oct 17;3(2):e002683. doi: 10.1136/bmjph-2025-002683 (PMC12542741; doi:10.1136/bmjph-2025-002683)
Supplement: online supplemental file 1 [file bmjph-3-2-s001.docx]

**Exploring the perspectives and experiences of food insecure adults who also experience tobacco dependence: Topic guide**

1. Current Life circumstances; stressors etc
   1. Can you walk me through a typical day in your life?
   2. What is your current living situation? Who do you live with and your relationship to them?
   3. What things do you most worry about? What makes you feel stressed or anxious?
      1. How do you cope with this?
2. Main Questions
   1. I know you mentioned you smoke, could you tell me a little bit about what a typical day of smoking looks like?
      1. What is your general pattern of smoking in a typical day? What was yesterday like? (i.e., when do you have your first cigarette?)
      2. How long have you been smoking? Why did you start smoking?
      3. How many cigarettes do you smoke per day?
      4. Do you limit the number of cigarettes you smoke? If so, why?
      5. Does anyone else in your house smoke?
   2. What do you like about smoking? What do you not like about smoking?
   3. I wonder do you mind sharing a bit about where you buy cigarettes and how much they cost you each week?
      1. How do cigarettes fit into your budget? How do you pay for your cigarettes?
      2. Where do you purchase tobacco products (i.e., the corner shop; supermarket; illegal)
   4. Have there ever been times you found yourself not able to buy as many cigarettes or smoke as much as you would have liked?
      1. Are there any changes to how much or how often you smoke based on how much money you have?
      2. How would you make your decisions around how much to smoke and how many cigarettes to buy?
   5. How does smoking affect your food choices, what you eat and when you eat?
      1. i.e., what do you eat and when?
   6. Current and past experiences of food insecurity
      1. Can you tell me more about your current food situation?
   7. What services/organisations have helped you with food (i.e., food banks, pantries, soup kitchens)?
      1. How do you find these organisations?
      2. What help/food items do they provide?
      3. Are you able to share with me any other help/advice these organisations provide you, or that you know they provide?
   8. How do you cope with having reduced food? Are there strategies you implement to help make your food last longer?
   9. How do you manage your food budget alongside spending on tobacco products?
      1. Can you tell me about a time when you had to make a choice between purchasing tobacco and food? How did you make that decision?
   10. Are there food items you go without to ensure you can afford tobacco?
   11. Can you talk me through a bit about your budgeting (weekly/monthly)?
       1. What do you do to make ends meet?
   12. Are there any strategies you use when shopping for food? i.e., shopping at multiple stores, buying cheaper own brand items, bulk buying etc
   13. How do you prioritise what gets paid? (i.e., bills; tobacco; food etc)
       1. What are the important bills/items that you pay for
   14. How do you think experiencing both food insecurity and tobacco dependence impacts your life and how you maintain your health and wellbeing?
   15. How do you think that smoking impacts your food insecurity? How do you think that your food insecurity impacts your smoking?
   16. Do you think smoking and food insecurity are linked in anyway?
       1. If so, how are they linked
   17. Can you tell me a little bit about stigma and food insecurity/smoking?
3. Health and smoking cessation services
   1. What are your thoughts about quitting? Have you tried to quit before? Can you tell me more about the circumstances around that? What support would you want for quitting?
   2. Can you talk me through what stop smoking services you know of?
      1. If you wanted to stop smoking – how would you do it? Where would you go for help?
   3. How does smoking affect your health?
      1. Do you consider yourself to have good health/poor health etc
      2. Does smoking affect your health in a positive or negative way
   4. What do you perceive the negative effects smoking has on health?
   5. What do you perceive the positive effects smoking has on health?
   6. How does reduced access to food affect your health?
   7. If you were to create a stop smoking service, what would you like it to look like?
      1. How would you like it operate?

Thank you so much for taking the time to talk to me today – I appreciate all you have told me. I just have one more question to ask you,

1. Is there anything we haven’t discussed today that you would like to tell me?
